# Color opponent receptive fields self-organize in a biophysical model of visual cortex via spike-timing dependent plasticity

**DOI:** 10.3389/fncir.2014.00016

**Published:** 2014-03-12

**Authors:** Akihiro Eguchi, Samuel A. Neymotin, Simon M. Stringer

**Affiliations:** ^1^Oxford Centre for Theoretical Neuroscience and Artificial Intelligence, University of OxfordOxford, UK; ^2^Department of Physiology and Pharmacology, Downstate Medical Center, State University of New YorkNew York, NY, USA; ^3^Department of Neurobiology, Yale University School of MedicineNew Haven, CT, USA

**Keywords:** brain modeling, visual cortex, neocortex, color, color selectivity, self-organizing color maps, self-organizing feature maps, STDP

## Abstract

Although many computational models have been proposed to explain orientation maps in primary visual cortex (V1), it is not yet known how similar clusters of color-selective neurons in macaque V1/V2 are connected and develop. In this work, we address the problem of understanding the cortical processing of color information with a possible mechanism of the development of the patchy distribution of color selectivity via computational modeling. Each color input is decomposed into a red, green, and blue representation and transmitted to the visual cortex via a simulated optic nerve in a luminance channel and red–green and blue–yellow opponent color channels. Our model of the early visual system consists of multiple topographically-arranged layers of excitatory and inhibitory neurons, with sparse intra-layer connectivity and feed-forward connectivity between layers. Layers are arranged based on anatomy of early visual pathways, and include a retina, lateral geniculate nucleus, and layered neocortex. Each neuron in the V1 output layer makes synaptic connections to neighboring neurons and receives the three types of signals in the different channels from the corresponding photoreceptor position. Synaptic weights are randomized and learned using spike-timing-dependent plasticity (STDP). After training with natural images, the neurons display heightened sensitivity to specific colors. Information-theoretic analysis reveals mutual information between particular stimuli and responses, and that the information reaches a maximum with fewer neurons in the higher layers, indicating that estimations of the input colors can be done using the output of fewer cells in the later stages of cortical processing. In addition, cells with similar color receptive fields form clusters. Analysis of spiking activity reveals increased firing synchrony between neurons when particular color inputs are presented or removed (ON-cell/OFF-cell).

## Introduction

It has long been known that many neurons in primary visual cortex (V1) are tuned to exhibit preference to particular simple oriented line segments, forming orientation maps that capture the preferred orientation of neurons across the cortical surfaces (Hubel and Wiesel, 1962). Similarly, clusters of color-selective neurons in areas V1/V2 have been reported, as mapped with optical imaging and electrophysiological recordings (Landisman and Ts'O, 2002; Friedman et al., 2003; Xiao et al., 2003; Lu and Roe, 2008; Salzmann et al., 2012). While several computational studies have been conducted to explain the emergence of the orientation map (Somers et al., 1995; Choe and Miikkulainen, 1998; Paik and Ringach, 2011), only a few have been done over such patchy distribution of color selectivity within an area of V1/V2 (Bednar et al., 2005; Rao and Xiao, 2012). Barrow et al. (1996) have proposed a model for the formation of cortical blobs, regions in primary visual cortex that are densely stained by cytochrome oxidase (CO) (Livingstone and Hubel, 1984), using the Hebbian learning rule. This model reproduces receptive fields of neurons inside and outside CO blobs, and the results showed that neurons outside the blobs are selective for orientation while neurons inside the blobs are selective for color. However, the spatial organization of a large number of color-selective areas was not studied in their model. In this paper, we investigate the emergence of the spatial organization of color preference maps by developing a hierarchical neural network model that reflects anatomically faithful processing pathways and projections.

Physiological studies have shown that color information is first represented by the activity of specific types of photoreceptors and transmitted along specific fibers in the optic nerve (Komatsu, 1998). Visual signals leaving the eyes then reach the primary visual cortex via the lateral geniculate nucleus (LGN). LGN has multi-layered organization, and different color information is coded at specific layers (Chatterjee and Callaway, 2003). Although actual neural processing is not known, Komatsu and Goda (2009) theorized that a two-stage model can explain the transformation of color signal that takes place between photoreceptors and V1, resulting in forming the color selective neurons. At the first stage, signals from color opponent neurons are linearly summed with various combinations of weights, with the results rectified. This information is then propagated to neurons in the second stage where a further linear summation and rectification is performed.

Rao and Xiao (2012) have recently started investigating similar principles in computational simulations and successfully produced maps of orientation and color selectivity using anatomically realistic projections incorporating two color opponent channels and a luminance channel. However, this model used rate-coded neurons, which do not convey the precise times of action potentials or spikes emitted by cells. Various physiological studies have indicated that spiking dynamics can be important for the simulation and information processing (Sugase et al., 1999; Freiwald and Tsao, 2010). Although our current model does not investigate orientation selectivity, one of the aims of our study is to expand the focus in previous research (Bednar et al., 2005; Rao and Xiao, 2012) to see if it is possible to observe the spatial organization of color preference maps and spike-timing related phenomena such as ON/OFF selectivity using more physiologically realistic Hodgkin–Huxley (HH) neuron models via Spike-Timing Dependent Plasticity (STDP).

Many neural networks are implemented with rate-coded neuron since it is observed that the mean firing rates of sensory neurons are correlated with the intensity of the encoded stimulus feature. For example, it is widely viewed that the information sent to the visual cortex by the retinal ganglion cells are encoded by the mean firing rates of spike trains generated with a Poisson process. A theoretical study conducted by Rullen and Thorpe (2001) showed that rate codes are optimal for fast information transmission but cannot account for the efficiency of information transmission between the retina and the brain; however, temporal structure of the spike train can be efficiently used to maximize the information transfer rate. This could therefore be an important feature that contributes to the development of neurons tuned to specific features.

Another benefit of our approach is that the precise firing times of spiking HH neurons allow investigating the temporal dynamics of information processing. Such investigations could include determining the role of temporal processing of C1, C2, and L channels in LGN (Chatterjee and Callaway, 2003), and selective representation of different stimuli by neuronal population synchronization (Evans and Stringer, 2013). In addition, spiking neurons allow incorporation of biologically plausible learning rules, such as STDP. A number of experiments (Markram et al., 1997; Bi and Poo, 1998) have reported that synaptic strength changes depending on presynaptic and postsynaptic spike time, and this mechanism has been extensively studied from a theoretical point of view (Gerstner et al., 1996; Abbott and Nelson, 2000).

Meanwhile, similar to the orientation maps and color maps, physiological studies have shown that various brain areas manifest a small-world structure, characterized by the presence of highly clustered neurons (Yu et al., 2008), and the factors leading to this organization have been investigated in several theoretical works (Shin and Kim, 2006; Kato et al., 2007, 2009; Basalyga et al., 2011). In the present study, we were particularly interested in whether such small-world structures could evolve from a network whose weights were initialized randomly, after learning with natural images.

We speculated there would be difficulty in the development of such cells since the representation of color is more complex than oriented bars. However, with this model, we hypothesized that the response patterns of neurons in the output layer (layer 5 of V1) would develop heightened responses to specific colors solely due to learning taking place during exposure to multiple image patches extracted from natural images of indoor scenes used in Quattoni and Torralba (2009), as a result of integrating different color opponent signals that occurred at different levels of the network. We also hypothesized that the learning would allow for a distribution of neurons that were tuned to similar color input with spatial clustering, where neurons within the cluster had heightened synaptic weights, relative to neurons outside of the cluster.

## Materials and methods

### Model

#### Architecture

The model is composed of nine layers of neurons which are organized into five hierarchical areas: photoreceptor layers (R, G, B), lateral geniculate nucleus (LGN) layers (L, C1, C2), V1 layer 4 (L4), V1 layer 2/3 (L2/3), and V1 layer 5 (L5). The dimensions of each layer are shown in Table 1, and the total number of neurons is thus 5700.

**Table 1:** **Dimensions of each layer**.

**Layer**	**Dimensions (number of cells)**
V1 layers (L4, L2/3, L5)	30 × 30
LGN layers (C1, C2, L)	30 × 30
Photoreceptor layers (L, M, S)	10 × 10

**Figure 1:**
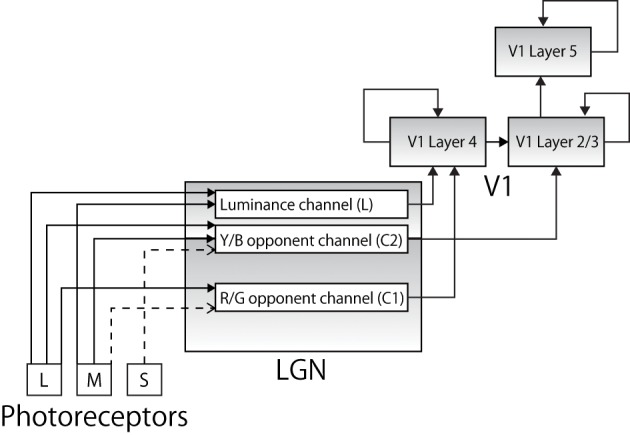
**The pathways along which color information from the photoreceptors is conveyed to cortical area V1 (solid lines represent excitatory connections and broken lines represent inhibitory connections)**. Each color input is represented by a specific combination of corresponding firing frequencies of trichromatic cones. Each signal is projected to anatomically appropriate layer in LGN layers forming a luminance channel and two color opponent channels. The output is then projected to appropriate layer in V1.

Each color input presented to the network is first decomposed into an RGB representation (range: 0–1) in digital images to be consistent with the trichromatic color vision in primates as a result of S, M, and L cones (Rowe, 2002) (Figure 1). The degree of each input is represented as different spiking frequencies of photoreceptors with 10% of random noise. To be consistent with physiology, a stimulus that a human would perceive as red activates the green channel very strongly as well. The frequency of each cone is determined as follow:

- *S*_freq_ = 40[Hz] × *B*
- *M*_freq_ = 40[Hz] × (*G* + *R* × 0.7 + *B* × 0.25)/(1 + 0.7 + 0.25)
- *L*_freq_ = 40[Hz] × (*R* + *G* × 0.7 + *B* × 0.25)/(1 + 0.7 + 0.25)

Specific combinations of the decomposed color signals are then projected to cells in LGN. The projections reflect the physiological findings that reported different characteristics in different layers of LGN (Shapley et al., 1981). Specifically, as later studies revealed, different layers of LGN receive different visual information via optic nerves and show different functionality, forming a luminance channel (L) and two opponent color channels, comprising red–green (C1) and blue–yellow (C2) channels as follows (Casagrande, 1994; Goda et al., 2009; Rao and Xiao, 2012):

- Magnocellular (MC) pathways: luminance channel *L* = *R* + *G*
- Parvocellular (PC) pathway: red/green opponent channel *C*1 = *R* − *G*
- Koniocellular (KC) pathway: blue/yellow opponent channel *C*2 = (*R* + *G*) − *B*

Physiological studies also report that while the MC and PC pathways project their output to V1 L4, the KC pathway terminates in V1 L2/3 (Chatterjee and Callaway, 2003), and many neurons in L2/3 project excitatory connections to the neurons in V1 L5 (Douglas and Martin, 2007). Our model incorporates this anatomical architecture (Figure 1). Physiological evidence indicates that there is heavy feedback from V1 to LGN (from layer 6) and the thalamic reticular nucleus is involved in both the feed-forward and feedback pathways, and data also suggests that retinal ganglion cells have widely different spatial extent; however, these are beyond the scope of this paper and are not explicitly modeled.

#### Synaptic connections

Convergent connections are established to each neuron from a topologically corresponding region of the preceding layer, leading to an increase in the receptive field size of neurons through the visual processing areas, which reflects the known physiology of the primate ventral visual pathway (Pettet and Gilbert, 1992; Freeman and Simoncelli, 2011). While synaptic weights between the photoreceptor layers and LGN layers are kept static, the weights of other feed-forward connections are learned through visually guided learning.

**Figure 2:**
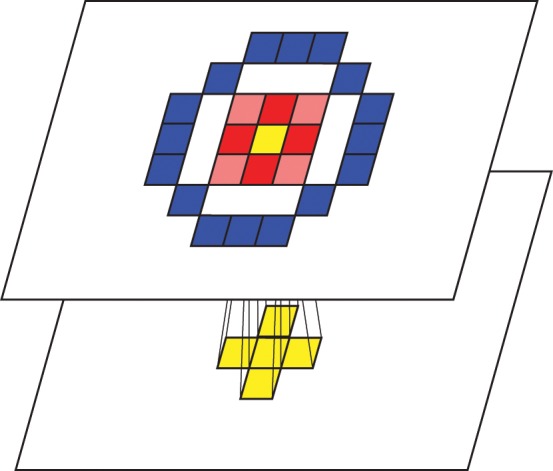
**Conceptual visualization of the inter/intra layer connectivities**. Activations of adjacent cells in the preceding layer are transmitted to a topologically corresponding cell in the following layer. Tiles filled with red represent cells that receive excitatory lateral connections while tiles filled with blue represent cells that receive inhibitory lateral connections, forming a Mexican-hat spatial profile.

Each feed-forward connection requires a 1 ms delay for signal transmission. Each neuron also establishes lateral short-range excitatory connections and long-range inhibitory connections, forming a Mexican-hat spatial profile (Figure 2). Whether this kind of lateral connectivity exists at the anatomical level is debatable (Martin, 2002; Kang et al., 2003; Hopf et al., 2006; Adesnik and Scanziani, 2010), since a detailed microcircuitry map at the neuron-to-neuron level is not currently available. However, we incorporated this architecture to (1) be consistent with a previous model by Rao and Xiao (2012) and (2) to abstract the function exhibited by this kind of architecture (Kang et al., 2003; Neymotin et al., 2011b). Further experimental work that details the wiring of cortical microcircuitry may reveal whether these considerations were justified (Alivisatos et al., 2013). The synaptic delay is 1 ms for the excitatory connections and 4 ms for the inhibitory connections.

#### Learning mechanism (STDP)

**Figure 3:**
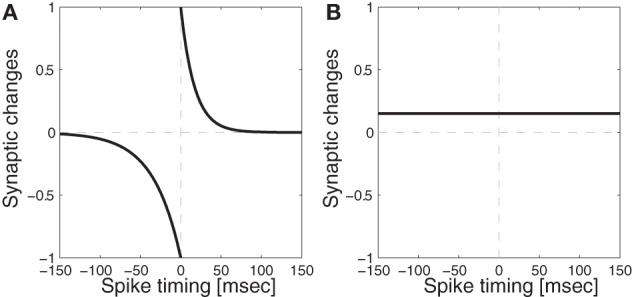
**Synaptic modification functions with/without Spike-Timing Dependent Plasticity (STDP)**. **(A)** Function with STDP: temporal windows for depression (*d*_tau_ = 34 ms) and potentiation (*p*_tau_ = 17 ms) used for spike-timing dependent plasticity where the equation is given in Equation (1) **(B)** Function without STDP: the synaptic weights are potentiated whenever both pre and post synaptic neurons become activated during the training time for 300 ms.

While synaptic weights at the connections between photoreceptor layers and LGN layers were fixed, weights in all the other feed-forward connections were plastic. Each synaptic weight in the model was learned using STDP, where Long-term potentiation (LTP) is caused if the pre-synaptic spike precedes the postsynaptic spike, and Long-term depression (LTD) is caused if the spike timing is in the opposite order. The degree of the modification depends on how close the two spikes are in time (Bi and Poo, 1998) as follows:

(1) $$\Delta w=\{\begin{array}{cc} \text{LR}\times \exp\left(\frac{-(t_{\text{post}}-t_{\text{pre}})}{p_{\text{tau}}}\right) & if(t_{\text{post}}-t_{\text{pre}})>0\\ -\text{LR}\times \exp\left(\frac{t_{\text{post}}-t_{\text{pre}}}{d_{\text{tau}}}\right) & if(t_{\text{post}}-t_{\text{pre}})<0 \end{array}$$

where LR is a learning rate, *t*_pre_ is the time when presynaptic cell becomes activated, *t*_post_ is the time when postsynaptic cell becomes activated, and *p*_tau_/*d*_tau_ controls the range of the influence. The curve generated by this function is show in Figure 3. Weights are originally randomly assigned within a fixed range, and after every iteration, weights in the same layers are normalized so that the mean of all the values are always kept in the middle of the pre-specified range, and also to prevent runaway excitation (Neymotin et al., 2011a, 2013; Rowan and Neymotin, 2013). Neurophysiological evidence for synaptic weight normalization is provided by Royer and Paré (2003).

#### Neuron model

Model neurons utilized the standard parallel conductance model with Hodgkin–Huxley dynamics for generating action potentials. Neurons consisted of a single compartment (diameter of 30 μm, length of 10 μm, axial resistivity of 100 Ωcm). The rate of change of a neuron's voltage (V) was represented as −*C*_*m*_ $\frac{dV}{dt}$ = *g*_pas_(*v* − *e*_leak_) + *i*_syn_ + *i*_*Na*_ + *i*_*K*_, where *C*_*m*_ is the capacitive density (10 μF/cm^2^), *i*_syn_ is the summed synaptic current, and *i*_*Na*_ and *i*_*K*_ represent the *Na*^+^ and *K*^+^ currents from the Hodgkin–Huxley channels. *g*_pas_ represents the leak conductance (0.001 nS), which was associated with a reversal potential, *e*_leak_, of 0 mV.

Synapses were modeled using an instantaneous rise of conductance, followed by exponential decay with specified time-constant, τ. For excitatory synapses, we utilized AMPA synapses (τ = 5 ms, *e*_rev_ = 0 mV), while for inhibitory GABA synapses (τ = 10 and *e*_rev_ = −80). Synaptic currents followed *i*_syn_ = *g*(*v* − *e*_rev_), where *v* is the membrane potential, and *e*_rev_ is the reversal potential associated with the synapse.

#### Software

Simulations were run using the NEURON simulation environment with the Python interpreter, multithreaded over 16–32 threads (Hines and Carnevale, 2001; Carnevale and Hines, 2009; Hines et al., 2009). Simulation is posted on ModelDB (https://senselab.med.yale.edu/ModelDB/ShowModel.asp?model=152197) (Hines et al., 2004). Simulations were run on Linux on a 2.93 GHz 16-core Intel Xeon CPU X5670. A 300 ms simulation ran in approximately 30 s.

### Data analysis methods

#### Clustering

In order to quantify the degree of clustering of the activations in the network, a clustering coefficient *C* is calculated based on the responses among different color inputs at every training iteration as follows (modified from Kato et al., 2007):

(2) $$C=\frac{1}{n\text{Cells}\times n\text{Stims}}\sum_{s=1}^{n\text{Stims}}\sum_{i=1}^{n\text{Cells}}C_{s,i}$$

(3) $$C_{s,i}=\frac{\sum_{l=1}^{k_{s,i}}\sum_{m=l+1}^{k_{s,i}}\left({\text{FR}}_{s,l}\times {\text{FR}}_{s,m}\right)}{{}_{ki}{\text{C}}_{2}}$$

where *n*Cells is the number of neurons in a network; *n*Stims is the number of stimuli during the testing; FR_*s,i*_ is the firing rates of the cell *i* when exposed to a stimulus *s*; *k*_*i*_ sets the nearby neurons from the *i*-th neuron for the analysis. We use 9 (3 × 3) for the *k* value.

#### Single-cell information

A single cell information measure was applied to individual cells to measure how much information is available from the responses of a single cell about which color input is present. The amount of color specific information that a certain cell transmits is calculated from the following formula:

(4) $$I(s,\vec{R})=\sum_{r\in \vec{R}}P(r|s){\text{log}}_{2}\frac{P(r|s)}{P(r)}$$

Here *s* is a particular color and $\vec{R}$ is the set of responses of a cell to the set of color stimuli, which are composed of eight colors slightly varied the RGB values of original color by ±1%. This is based on the assumption that the same set of tuned cells will still respond to slightly variant colors and is to well differentiate the tuned cells from randomly responding cells. The maximum information that an ideally developed cell could carry is given by the formula:

(5) $$\text{Maximum cell information}={\text{log}}_{2}(n\times p)\text{ bits}$$

As eight different sets of colors (combination of 0 and 1 for each RGB value) are used in this analysis, the maximum information could be carried in this analysis is 3.

#### Multiple-cell information

A multiple-cell information measure was used to quantify the network's ability to tell which stimulus is currently exposed to the network based on the set of responses, *R*, of a sub-population of cells, $\vec{C}$, as following formula with details given by Rolls and Milward (2000).

(6) $$I_{\vec{C}}\left(S,S^{\prime }\right)\;=\sum_{s,s^{\prime }}P\left(s,s^{\prime }\right){\text{log}}_{2}\frac{P\left(s,s^{\prime }\right)}{P(s)P\left(s^{\prime }\right)}$$

(7) $$P\left(s^{\prime }\right)\;=\sum_{s\in S}P\left(s^{\prime }|R_{\vec{C}}(s)\right)\times P\left(R_{\vec{C}}(s)\right)$$

(8) $$P\left(s,s^{\prime }\right)\;=P\left(s^{\prime }|R_{\vec{C}}(s)\right)\times P\left(R_{\vec{C}}(s)\right)$$

Here, *S* represents the set of the stimuli presented to the networks, and $\vec{C}$ defines the set of cells used in the analysis, which had as single cells the most information about which color input was present. From the set of cells $\vec{C}$, the firing responses *R*_$\vec{C}$_ (*R* = *r*(*c*)|*c* ∈ $\vec{C}$) to each color in *S* are used as the basis for the Bayesian decoding procedure as follows:

(9) $$P\left(s^{\prime }|R_{\vec{C}}\right)=\frac{P\left(s^{\prime }\right)\prod_{c\in \vec{C}}P\left(R_{c}(s^{\prime })|s^{\prime }\right)}{\sum_{s^{\prime \prime }\in S}P\left(s^{\prime \prime }\right)\prod_{c\in \vec{C}}P\left(R_{c}(s^{\prime \prime })|s^{\prime \prime }\right)}$$

(10) $$P\left(R_{c}(s)\right)|s^{\prime })=\frac{\sum_{t=1}^{n\text{Trans}}pdf\left(R_{c}(s,t),{\bar{R}}_{c}\left(s^{\prime }\right),SD_{c}\left(s^{\prime }\right)\right)}{n\text{Trans}}$$

where *n* Trans defines the number of possible transforms; in this case, similar but slightly different colors, and pdf computes the probability density function at firing response of a subset of cells when exposed to a stimulus *s* at *t*th transforms using the normal distribution with their mean and standard deviation.

## Results

The results described in this study used a network model trained with various small color image patches extracted from original natural images of indoor scenes used in Quattoni and Torralba (2009). The size of the photoreceptor layer in our model is 10 × 10 pixels while the size of original images was an average of 504.1 × 658.4 pixels (112 images). The training session consisted of 2000 iterations, where 2000 different 10 × 10 image patches were extracted from the set of images. This was designed as an abstraction of natural viewing, where eyes saccade, and the activation of photoreceptors corresponds to visual inputs bounded by their range of view.

### Learning produces spatial clustering

**Figure 4:**
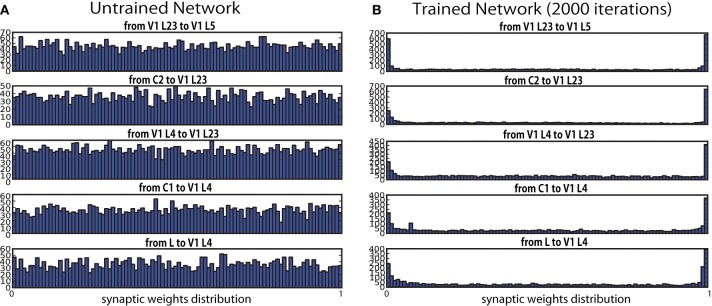
**Synaptic weight distribution at synapses before (0 iteration) and after (2000 iterations) the learning**. Weights are initialized randomly. After every iteration, connection weights between the two layers are normalized so that the mean of all the values are kept in the middle of the pre-specified range, and to prevent runaway excitation. The graphs show that the weights converged over the course of the training sessions. **(A)** Untrained network. **(B)** Trained network.

During the training, synaptic efficacy between each of two layers progressed from a uniform distribution at the initial state toward a binary distribution where only a limited number of synaptic connections were highly strengthened or weakened (Figure 4). This convergence toward an bimodal equilibrium state is consistent with other self-organizing modeling work with STDP (Song et al., 2000; Kato et al., 2009; Basalyga et al., 2011). Contrary, physiological studies have shown that synaptic weights tend to have unimodal distributions with a positive skew (Barbour et al., 2007). Barbour et al. (2007) raised a possible reconciliation with the bimodal distributions of modeling with such experimental data, given that the dendritic distribution of synaptic weights may have a wide range of values, due to electrotonic filtering effects. However, in order to explore this possibility, further investigation will be required.

**Figure 5:**
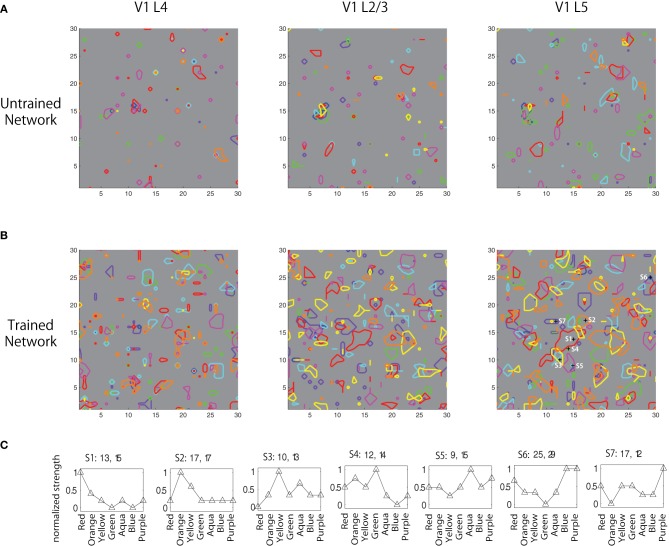
**Boundaries of peaks of firing counts for seven colors (red, orange, yellow, green, aqua, blue, and purple) (A) before, (B) after training, and (C) normalized firing activity of seven neurons in V1 L5 in response to the seven color inputs were plotted**.

Investigation into the firing count of each neuron to different color inputs shows that the weight convergence resulted in development of clustered responses in the networks (Figure 5). A comparison between the results with the weight distribution plots in Figure 4 shows that even though the average weight was kept constant, neuronal firing activity became more prominent and deviated after the training; it was sparse (average rate of 2.165 Hz with standard deviation of 0.874) prior to learning, but after 2000 iterations of 300 ms exposure to image patches extracted from natural indoor images, the network developed different clustered firing patterns of neurons (average rate of 3.966 Hz with standard deviation of 1.169) for eight different color inputs (red, orange, yellow, green, aqua, blue, purple, and pink).

In Figures 5A,B, the boundaries of peaks of firing counts for seven different colors (red, orange, yellow, green, aqua, blue, and purple) before and after training are plotted. The result shows that the training resulted in developing color selective clustered responses. Normalized firing activity of seven neurons in V1 L5 were recorded and plotted in Figure 5C. These results failed to show a clear spatial shift of the activation with gradual change of color inputs as reported in Xiao et al. (2003); however, the results revealed gradual changes of firing patterns according to changes of input colors, which is partially consistent with the physiological findings. This also shows that some cells show higher selectivity than others at responding to similar colors. This is likely due to the fact that the color representation takes a specific combination of three continuous values of RGB. Depending on the trained weights, activations of some neurons may only be influenced by one or two of the three values, and the activation patterns also vary due to different combinations of those values and influences from other nearby neurons.

**Figure 6:**
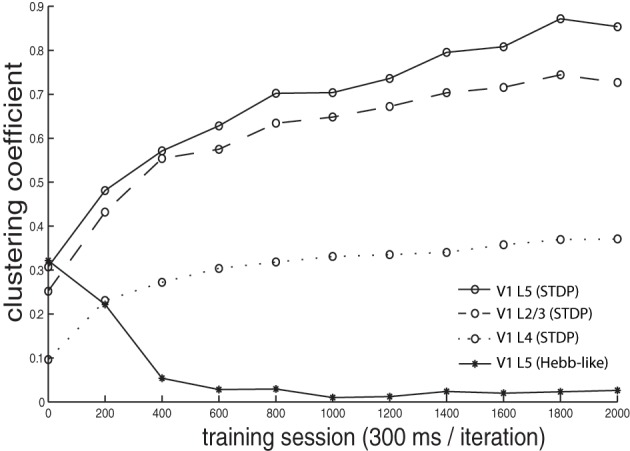
**Clustering coefficient dynamics during the training**. The clustering coefficients *C* of V1 L4, L2/3, and L5, where the networks were trained with STDP, were calculated by the equation given in Equations (3, 3) and plotted by dotted line, dashed line, and solid line with circle markers, respectively. Additionally, the clustering coefficients of V1 L5 when the network was implemented with Hebb-like rule is plotted by the line with asterisk markers. The result demonstrates that the networks trained with STDP rule gradually increases clustering coefficients as training proceeds while the network trained with Hebb-like learning rule remains relatively low clustering coefficient.

We calculated a clustering coefficient [*C*; Equations (3, 3)] to assess the effectiveness of training in producing spatial clustering within the network. Figure 6 shows *C* of V1 L4, V1 L2/3, V1 L5, as well as of V1 L5 trained with Hebb-like learning rule, plotted as a function of training iteration. The result demonstrates that the networks trained with STDP rule gradually increases clustering coefficients as training proceeds while the network trained with Hebb-like learning rule remains relatively low clustering coefficient.

The emergence of clustering may be explained by the lateral excitatory connections described in section 2.1.2. When a specific neuron becomes activated, the signal is propagated to the neighboring neurons making them more likely to become activated as well. Once the neighboring cell reaches a threshold and becomes activated, synaptic connections convergent onto the cell from recently activated cells in the preceding layer become strengthened via STDP. Repetitions of this process are likely to be the cause of the development of the clustered responses of cells. This phenomenon should be prominent only among nearby cells because of lateral propagation delays and long-range lateral inhibition.

The precise temporal dependence of STDP is crucial for the clustering learning process. Activation of neurons are laterally propagated within layers but with a specified delay. Therefore, temporal differences of the activations between the source in the preceding layer and the targets in the following layers become large as the signal is propagated. As a result, the degree of LTP decays as the differences become large, and LTD is turned on if the post-synaptic activation timing becomes closer to the next pre-synaptic activation, thus forming the distinct clustering responses in the networks.

In order to confirm the importance of spike-timing in forming color receptive field clustering, we ran a control simulation, using a Hebbian plasticity synaptic learning rule, which does not take into account the timing of pre- and post-synaptic neuronal spiking (Figure 3B). After learning with this Hebbian plasticity rule, the clustering coefficient value remained low (Figure 6 lines with asterisks) relative to the results in the network trained with STDP. This underlines the importance of STDP in developing clustering in our model.

In addition, our model shows that the clustering coefficient in higher layers tended to be larger. This observation makes us expect information to gradually change in the different layers, and this assumption has been confirmed in the next section.

### Selectivity of the responses

In order to identify how the learned connectivity shaped output neuron sensitivity to stimuli, the techniques of Shannon's information theory were employed (Rolls and Treves, 1998). If the responses *r* of a neuron carry a high level of information about the presence of a particular color stimulus *s*, this implies that the neuron will respond selectively to the presence of that color. Two information measures were used to assess the ability of the network to develop neurons that are selective to the presence of a particular color by measuring single cell and population information (see sections 2.2.2, 2.2.3). Since eight different sets of colors (red, orange, yellow, green, aqua, blue, purple, and pink) are used in this analysis, the maximum information carried in this analysis is 3 bits.

**Figure 7:**
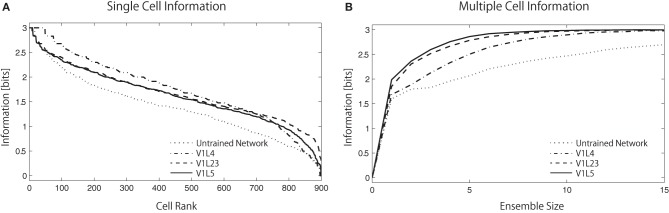
**Single-cell/Multiple-cell information analysis of V1 L5**. Solid lines represents trained networks while dotted line represents naive network. **(A)** The single cell information measure are plotted in rank order according to how much information they carry. The result show that the maximum information each cell carry drops rapidly in the naive network while most of the cells in the trained network carry relatively higher amount of information. **(B)** The multiple cell information measures are plotted according to the number of cells used in the analysis to visualize the mutuality of the responses. The information that the trained network carries reaches maximum mutual discriminability with 3 bits of information with around 8 neurons while the information that the naive network carries does not reach this point with 15 neurons. This result also shows that fewer neurons are required to represent all stimuli in the higher layers, as the information measure improves from L4 → L2/3 → L5.

Figure 7A shows the single cell information analysis as plotted in rank order according to maximum information each cell carries for a specific stimulus. The results compare the information distribution of each layer in the trained network and of the final layer (V1 L5) in the untrained and trained network. The results demonstrate that neurons in the trained network generally carry more single-cell information.

While useful in assessing the tuning properties of a particular neuron, the single-cell information measure cannot provide mutuality of the responses; if all cells learned to respond to the same color input (according to the single-cell measure) then there would be relatively little information available about the whole set of color stimuli *S*. To address this issue, we used a multiple-cell information measure, which assesses the amount of information that is available about the whole set of color inputs from a population of neurons (see section 2.2.3).

In Figure 7B, the multiple cell information measures are plotted according to the number of cells used in the analysis. The result shows that the trained network conveys more color specific information than the untrained network. More interestingly, we found that the amount of color specific mutual information reaches a maximum with fewer neurons in the higher layers: 13 neurons in L4, 10 neurons in L2/3, and 8 neurons in L5. This analysis indicates that estimations of the input colors can be done using the output of fewer cells in the later stages of cortical processing.

More precisely, the total amount of mutual information (across a layer) can not increase through further processing as the Data Processing Inequality (DPI) states—it can only be preserved or lost. In other words, if all the information from all cells in each of the two layers was added up, it will decrease in the higher layer. However, our specific information measure explained in section 2.2.3 can increase for particular cells, as they become more selective throughout the layers. In this case, some of that information has shifted into different cells, and so all stimuli can now be represented with fewer neurons, allowing for fewer required cells to convey maximum information. Our information measure therefore improves, showing that the cells are becoming more tuned, even though the total information in the layer has decreased.

### ON- and OFF-cells

**Figure 8:**
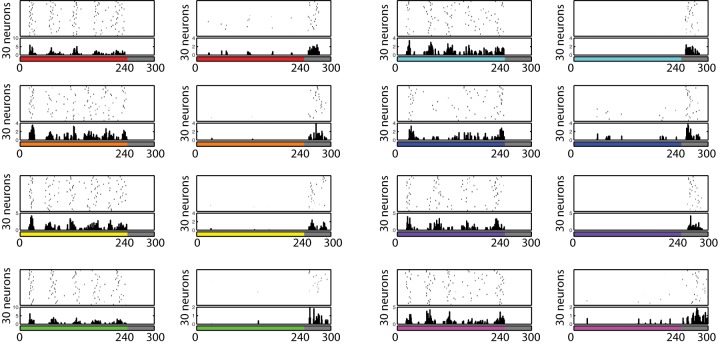
**Firing activity of 30 neurons in V1 L5, which responded vigorously when color input is presented or removed, from each experiment**. The color bars under each raster plot represent times at which colors are presented to the neurons (each color is presented for 240 ms and removed). From these results, we found that many neurons exhibit the characteristics of ON/OFF-cells in the trained network.

The firing pattern of each cell in response to turning a stimulus ON and OFF was also investigated. During this testing procedure, eight different colors (red, orange, yellow, green, aqua, blue, purple, and pink) are presented for 240 ms, followed by 60 ms of no visual input presentation, and the voltage level of each neuron is recorded. In order to find if any neuron developed ON/OFF sensitivities during training with similar properties to those found in V1/V2 *in vivo* (Michael, 1978; Friedman et al., 2003), from each recorded voltage dynamics, the 30 neurons which responded the most during the first 60 ms and the last 60 ms were selected to be plotted in Figure 8. Similar to the physiological findings, we found both ON- and OFF-cells for each different color input, where populations of neurons showed a burst of firing just after a presentation or removal of a color input.

**Figure 9:**
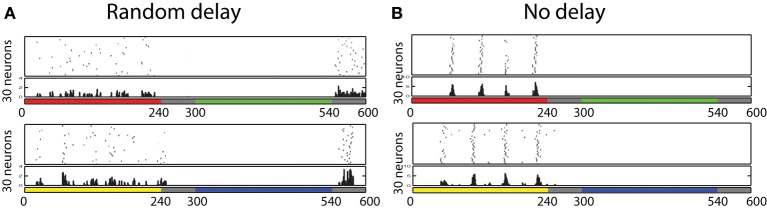
**Firing responses of neurons in the V1 L5, which shows temporal color opponent ON/OFF responses**. Figure on the top shows 30 neurons that responds highly when color red is presented and when color green is removed. Figure on the bottom shows 30 neurons that responds highly when color yellow is presented and when color blue is removed. This result shows that in the trained network, we found Yellow-ON/Blue-OFF cells and Red-ON/Green-OFF cells as reported in physiological experiments. **(A)** Random delay. **(B)** No delay

Also, further analysis revealed that some of those cells displayed the temporal color opponent property as reported in Friedman et al. (2003). Figure 9A shows two types of such cells: Red-ON/Green-OFF cells and Yellow-ON/Blue-OFF cells. In order to find such cells, we first identify 100 cells that show Red-ON (or Yellow-ON) property, and then chose 30 cells from the subset that show Green-OFF (or Blue-OFF) property.

Neurons in the layers are exposed to different colors in natural images during the training, so the development of ON-cells which exhibit specific responses to specific inputs can be explained with standard feed-forward competitive learning principles (Rolls and Treves, 1998). In contrast, the development of OFF-cells are due to the lateral inhibitory connections emitted by ON-cells: suppose there are ON-cells that were tuned to the color red. If red is presented to the network, these ON cells become activated making surrounding cells that receive inhibitory synaptic connections from the ON cells less likely to become activated. When the color input is removed, ON-cells stop activating. As a result, the surrounding cells are no longer suppressed by the ON-cells, demonstrating their being OFF-cells.

**Figure 10:**
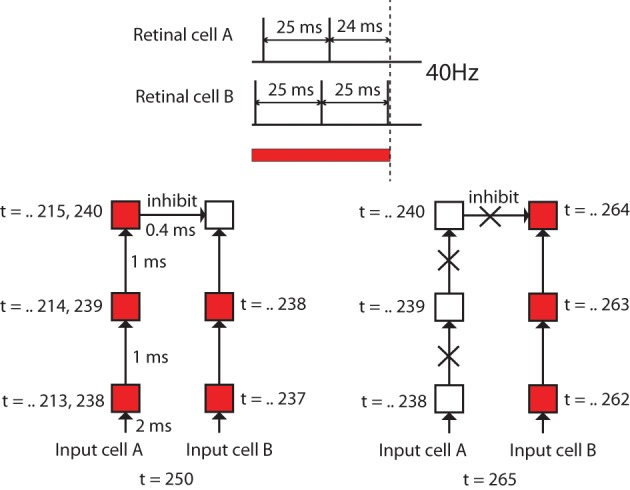
**Diagrams to explain the speculated cause of the differences in durations of firing activities of different neurons after removal of color inputs**. In this experiment, the maximum activation frequency of input cells was set to 40 Hz, and different input cells have different delays in the firing timings. Firing activation timing of input cell A is 1 ms later than another input cell B. This means there are 24 ms differences in the last activation before the removal of the color input. This difference will result in giving a chance for the OFF-cell that receives most of the inputs from the input cells such as B to become activated after an ON-cell that happens to receive most of the inputs from the input cells such as A stops releasing inhibitory signals.

However, the question is where the OFF-cells receive excitatory input to enable them to remain activated after the removal of the color input. In other words, there should be some mechanism where ON-cells immediately stop receiving excitatory input while OFF-cells keep receiving excitatory input, even after the removal of the color input. This may be caused by the differences in firing timing of different input cells as explained in Figure 10.

In our model, the maximum activation frequency of input cells was set to 40 Hz (25 ms interspike interval), which is gamma oscillations which are widespread in the visual cortex. Also, different input cells have different randomly determined delays from the input cell receiving color input to its firing, which is reflected in their firing timings. As shown in Figure 10, suppose the spike timing of an input cell A is 24 ms earlier than another input cell B. This means that there is at most 24 ms difference between the final spike timing of cell A and the timing of cell B before the removal of the color input. This 24 ms difference will result in giving a chance for the OFF-cell that receives most of the inputs from the input cells such as B to become activated after an ON-cell that happens to receive most of the inputs from the input cells such as A stops activating inhibitory signals.

In order to confirm the importance of the delay for the development of such ON/OFF cells, we have also trained the same network without randomly determined delays from the input cell receiving color input to its firing timings. Figure 9B shows the firing activities of each 30 neurons selected by the same procedure used to find Red-ON/Green-OFF cells and Yellow-ON/Blue-OFF cells earlier. The results show that in the network that employed inputs without randomized delays, we failed to find Green-OFF and Blue-OFF cells within each subset of 100 Red-ON cells and Yellow-ON cells. This result indicates that the randomized delay plays an important role for the development of the OFF cells.

In animal V1, much of the ON and OFF component of the responses are thought to be inherited from similar properties of LGN and RGC cells. Therefore, we are not expecting that onset and offset transients arise in V1 alone. However, our results suggested the possibility of multiple mechanisms that impact the firing times of these cells.

## Discussion

In this study, we have developed a model of early visual processing of colors including the pathway beginning at photoreceptors and terminating in the fifth layer of V1. We have incorporated anatomically accurate projections of signals between layers and the biologically plausible learning of synaptic weights based on STDP using Hodgkin–Huxley models of neuronal dynamics.

We have successfully shown that the networks gradually develop clustered firing activity of neurons during training (section 3.1). Information analysis based on averaged firing rates of each neuron also confirmed development of neuronal color selectivity after the training (section 3.2). Our results also indicated that populations of neurons can provide reliable predictions of the input color presented to the retina. Interestingly, the color information measure by multiple-cell information analysis rises more rapidly with fewer cells from L4 → L2/3 → L5, suggesting that layered neocortical architecture may enable it to *boost* important information. We also found that if the synaptic weights in the network were learned via a Hebbian plasticity rule, the level of clustering coefficient remained low relative to the results in the network trained with STDP.

However, the question is why other models without STDP, including the model by Rao and Xiao (2012), show similar types of clustering merely due to Mexican-hat connectivity. One possibility would be that in many hierarchical unsupervised neural network models, each layer is trained separately in turn. This is important for synaptic connectivities in higher layers to be appropriately tuned. However, in our model, all the synaptic connectivities are learned simultaneously, which may be more realistic. The implication would be that STDP may allow a network to learn connectivities more flexibly without the traditional *greedy* method of teaching one layer at a time. We propose this hypothesis because adding another dimension of timing via STDP allows the synaptic weights to be dynamically updated in real-time whereas rate coded neurons depend on averaged firing rates within pre-specified time windows.

Furthermore, investigating neuronal voltage dynamics revealed the presence of both ON-cells and OFF-cells, which respond maximally immediately after presentation or removal of a particular color input. These results led us to hypothesize that the emergence of OFF-cells was caused by different spike timing delays from input cells (section 3.3).

The role of neuronal synchrony in color processing is still an open question particularly since our model demonstrates that information analysis based on firing rates can successfully predict the color input. However, while the network was trained with various color input in natural images, in this analysis, the network was tested only with eight clearly distinct colors, and in order to accurately decode the subtle differences between similar colors, synchrony and its timing may play an important role for the representations at least in our proposing mechanism. In addition, the importance of timing delays in the creation of ON/OFF cells suggests rate codes alone may not be sufficient in visual system development.

### Role of spike-timing delays in creating ON/OFF cells

**Figure 11:**
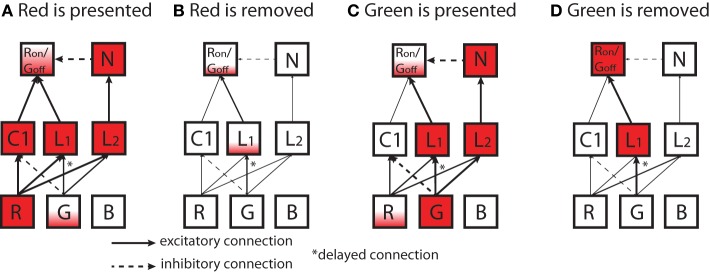
**Diagrams of a simplified network to explain emergence of ON/OFF-cells that is also combined with R/G opponency shown in Figure 9**. Each box represents a neuron. Inputs leading to the activation/inactivation of a *R*_ON_/*G*_OFF_ output cell is shown (**(A)** red is presented; **(B)** red is removed; **(C)** green is presented; **(D)** green is removed). Here, N represents the neighboring cell of the *R*_ON_/*G*_OFF_ output cell. In the intermediate layer, C1 is a cell in the Red/Green color opponent channel (R − G) and L is a cell in the luminance channel (R + G). In the input layer on the bottom, each R, G, B represent a photoreceptor. Neurons are filled in proportion to their activation level. Solid (broken) arrows represent excitatory (inhibitory) connections. A star placed next to an arrow means that the connection has delayed synaptic timing as discussed in Figure 10.

The mechanism of the emergence of OFF-cells due to spike timing delays allows us to propose a possible *in vivo* mechanism of the development of the ON/OFF-cell that is also combined with the R/G opponency shown in Figure 9. As shown in Figure 11, we suppose there is a simplified network that consists of three cells in the LGN layers and two cells in output layer (*R*_ON_/*G*_OFF_ cell and its neighboring cell N). In this schematic, LGN cells consist of a C1 (R/G opponent) cell and two L (monochrome) cells. In addition, one of the L cells, L_1_, has a delayed Green input (see details in Figure 10).

When the color red is presented to the network (Figure 11A), all three cells in the LGN become activated, and the *R*_ON_/*G*_OFF_ output cell that receives excitatory inputs from the C1 cell and one L cell (L_1_) becomes highly activated. When the red input is removed (Figure 11B), only L cells become slightly activated due to the delayed connection, which does not have a large influence on the *R*_ON_/*G*_OFF_ cell.

When Green color input is presented to the same network (Figure 11C), the L cells become activated. Subsequently, the N cell in the output layer that sends an inhibitory signal to the *R*_ON_/*G*_OFF_ cell becomes activated as well. Because of the inhibition, the *R*_ON_/*G*_OFF_ cell does not become highly activated even though it receives excitatory input from the preceding L_1_ cell. When the color input is removed (Figure 11D), the L_1_ cell that has the delayed connection from the Green cell is kept activated, which causes the *R*_ON_/*G*_OFF_ cell to become activated.

**Figure 12:**
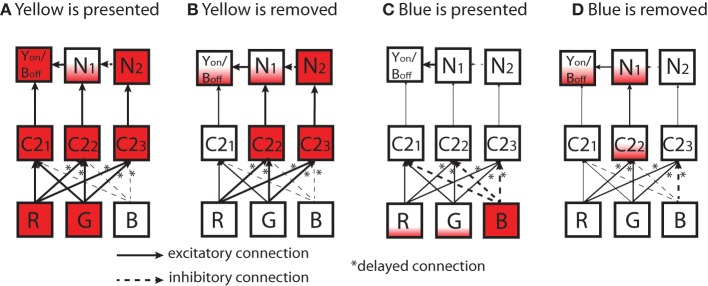
**Diagrams of simplified network to explain a possible mechanism of ON/OFF-cell that is combined with the Y/B opponency reported in Figure 9**. In the figure, each box represents a cell in the network. Inputs leading to the activation/inactivation of a *Y*_ON_/*B*_OFF_ output cell are shown: (**(A)** yellow is presented; **(B)** yellow is removed; **(C)** blue is presented; **(D)** blue is removed). In the output layer on the top, the target cell shown as *Y*_ON_/*B*_OFF_ in each box represents the ON/OFF target cell, N_1_ is a neighboring cell that sends excitatory connections to *Y*_ON_/*B*_OFF_, and N_2_ is a cell that sends excitatory connections to *N*_1_. In the intermediate layer, C2 is a cell in the Yellow/Blue color opponent channel [(R + G)−B]. In the input layer on the bottom, each R, G, B represent each photoreceptor. Cells are filled with a color to provide degrees of activations of different cells; partially filled box means it is only activated a small amount. All solid arrows represent excitatory connections while broken arrows represent inhibitory connections. A star placed next to an arrow means that the connection has a delayed synaptic timing as discussed in Figure 10.

Similarly, a possible mechanism of the ON/OFF-cell that is combined with Y/B opponency is provided in Figure 12. In the figure, we suppose a simplified network consists of three C2 (Y/B opponent) cells in the LGN layer and three cells in the output layer (*Y*_ON_/*B*_OFF_, N_1_, and N_2_). Each cell in the output layer receives excitatory input from one C2 cell (C2_1_, C2_2_, and C2_3_) cell). N_2_ cell establishes inhibitory connection to N_1_, and the N_1_ establishes an excitatory connection to the target cell, *Y*_ON_/*B*_OFF_. In this network, C2_2_ cell establishes the delayed connections discussed above (in Figure 10) from the R and G cells, and C2_3_ cell establishes delayed connections from all R, G, and B cells.

As shown in Figure 12A, when the color yellow is presented, all C2 cells become activated. As a result, the target cell, *Y*_ON_/*B*_OFF_, should become highly activated by receiving excitatory input from the preceding C2_1_ cell. In addition, the target cell *Y*_ON_/*B*_OFF_ receives some excitation from N_1_ cell. When the color input is removed (Figure 12B), due to the delayed connections, C2_2_ and C2_3_ cells are kept active for an interval, but not C2_1_. As a result, the target cell, *Y*_ON_/*B*_OFF_ would not get highly activated.

When the color blue is presented (Figure 12C), none of the C2 cells would become activated, leading to no activation of the target cell. On the other hand, when the color input is removed (Figure 12D), C2_2_ cell becomes activated to some degree due to the delayed connection from R and G with their weak activations caused by the color blue. This leads to the activation of N_1_ that establishes excitatory connection to the target cell, *Y*_ON_/*B*_OFF_. In this way, it is possible to provide a possible dynamical mechanism of ON/OFF-cells that involves color opponency.

**Figure 13:**
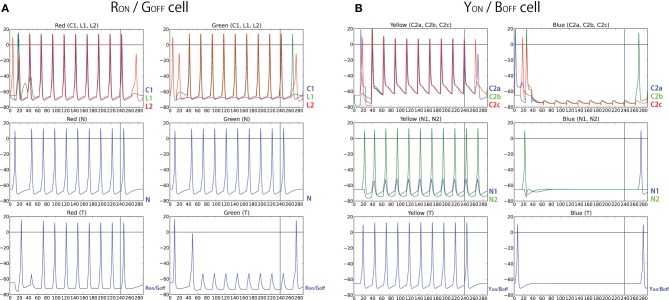
**(A)** Firing activity of each neuron in the simple network described in Figure 11. Left sub-panels show the activity when color input of red, RGB(1, 0, 0), is presented while right sub-panels show the activity when color input of green, RGB(0, 1, 0) is presented, both for 240 ms. In the figures on the top, the activity of C1, L_1_, and L_2_ are plotted with blue, green, and red color, respectively. The figure on the middle plots the activity of the neighboring cell, N, and the figure on the bottom plots the activity of the *R*_ON_/*G*_OFF_ cell. **(B)** Firing activity of each neuron in the simple network described in Figure 12. Left sub-panel shows the activity when color input of yellow, RGB(1, 1, 0), is presented while right-subpanel shows the activity when color input of blue, RGB(0, 0, 1), is presented, both for 240 ms. In the top panels, the activities of C2_1_, C2_2_, and C2_3_ are plotted with blue, green, and red color, respectively, and in the middle panels, the activities of N_1_ and N_2_ are plotted with blue and green color, respectively. The bottom panels display the activity of the *Y*_ON_/*B*_OFF_ cell.

In order to test the hypothesized architectures above, we have modeled the simple networks using the same set of neurons used in our computational model and recorded firing activity of each neuron for 300 ms (240 ms of color input presentation followed by 60 ms of no color input presentation) (Figure 13). The results show that the same target neuron exhibits characteristics of both *R*_ON_/*G*_OFF_, and *Y*_ON_/*B*_OFF_ firing activity. However, the result also showed that those responses are not observed immediately after the presentation or removal of the color input. In other words, there is still activity in ON-cells after the stimulus is turned off. Also, OFF-cells show responses when the stimulus is turned on. These effects are due to the transitional delay of signals. However, as shown in Figures 8, 9, the population activity shows a more clear ON/OFF response.

### Potential limitations

Although our model predicts that spike timing is important for the effective development of color selectivity, our model did not investigate development of orientation selectivity, which is known to coexist with color selectivity, as investigated in previous models (Barrow et al., 1996; Rao and Xiao, 2012). Therefore, in future work it will be important to model co-development of both color and orientation selectivity. A different limitation of our model is that the representation of color input was based on simplified input cells that detect digital RGB values. To investigate more realistic mechanisms of development, biologically-accurate architectures of the various types of retinal cells that are involved in the process should be implemented.

### Convergence of approaches

Our model of the early visual system displays convergence between the fields of computational neuroscience and artificial neural networks (ANNs). Computational neuroscience has traditionally attempted to understand neuronal dynamics by building models by using known biological detail without forcing an explicit engineered goal. ANNs, which emerged from the field of artificial intelligence, have stressed an approach that aims to develop systems displaying intelligence by constraining the system design to a specified goal, while taking inspiration from biological systems (Hinton et al., 2006).

Recent developments in ANNs, including *deep learning*, a technique drawing inspiration from neurobiology, have made significant progress in recent years (Hinton et al., 2006) improving performance on visual information processing (Lee et al., 2009). Progress has also been made by training recurrent neural networks to perform extremely well on difficult, specialized classes of problems, such as handwritten character recognition (Graves and Schmidhuber, 2008). Related developments have also started focusing on investigations into utilizing brain-inspired informatics to improve the intelligence of current technologies (Eguchi et al., 2013). However, currently, even the best machine learning algorithms have difficulty in matching human performance in recognizing arbitrary classes of complex visual stimuli. Basic research in neurobiology, combined with utilization of biological detail in computer models, is therefore needed to enable further improvements in machine learning. Improved understanding of how the brain circuitry represents and processes visual information may inspire new classes of visual processing algorithms. We have used this approach to design our model, which allows correlation of its neuronal dynamics with electrophysiological data, takes into account known neuroanatomy, and uses a biologically plausible learning rule (Markram et al., 1997), and therefore takes a step toward improved understanding of *in vivo* brain dynamics.

### Neocortical architecture

One of the basic goals of neuroscience is to elucidate the mechanisms by which the structure of the brain leads to its function (Shepherd, 2004). This depends on a careful study of neuroanatomy as well as functional measures *in vivo* (Weiler et al., 2008). The importance of changes in microcircuitry is underscored with experimental studies that have shown how alterations in cortical connectivity can lead to diseases, such as autism (Qiu et al., 2011). Since it is not possible to measure the state of all neurons it is important to combine computer modeling with known neurophysiological circuitry data (Lytton, 2008). Following this approach in our model allows us to make predictions on the function and development of several features observed in visual cortex *in vivo*.

Our model suggests that *in vivo*, the process of development of color clustering is more likely to initiate in earlier layers (L4) of V1. This may be testable via electrophysiological methods applied during different stages of development. Our model is also consistent with more general implications, suggesting that through a process of development, each layer of neocortex may learn to enhance important signals as they progress within the microcircuitry. Although initial synaptic weights in our model were randomly distributed, visual information and STDP allowed the feed-forward projections of the neocortex to learn the color information as the signals flowed in successive layers. In our model, the color information progressed from L4 → L2/3 → L5. Although L4 is the input layer into V1, the final output layer (L5) had the highest information content about the color stimuli. Further experiments will be needed to elucidate the role that individual layers play in shaping the information coding capacity of the neocortex.

Prior modeling (Stringer and Rolls, 2002; Rolls and Stringer, 2006; Dura-Bernal et al., 2012) and experiments (Hung et al., 2005) have shown the importance of the feed-forward architecture of the visual cortex ventral stream for object recognition. Although our work makes use of the feed-forward architecture of cortical areas, it also takes into account additional details of wiring, including recurrent connectivity. As more microcircuitry data becomes available, it will be possible to refine our model further (Alivisatos et al., 2013). Part of this process will involve combined experimental/computational approaches. For example, Hung et al. (2005) studied the ventral visual pathway with the aim of understanding how object recognition takes place by building pattern recognition algorithms that utilize inferotemporal cortex neuronal spiking information to assess both object category and identity. In the future it will be possible to extend our model to use similar techniques to quantify performance in object recognition that is based on accurate color processing.

### Conflict of interest statement

The authors declare that the research was conducted in the absence of any commercial or financial relationships that could be construed as a potential conflict of interest.
